# Efficacy of xenogeneic fresh and lyophilized amniotic membranes on the healing of experimentally induced full-thickness skin wounds in dogs

**DOI:** 10.1038/s41598-025-95023-9

**Published:** 2025-05-04

**Authors:** Kamal H. Hussein, Esraa Motiea, Manal T. Hussein

**Affiliations:** 1 Department of Surgery, Anesthesiology, and Radiology, Faculty of Veterinary Medicine, Assiut University, Assiut, 71526 Egypt; 2 Tissue Culture and Stem Cells Unit, Molecular Biology Researches & Studies Institute, Assiut University, Assiut, 71526 Egypt; 3 Department of Cell and Tissues, Faculty of Veterinary Medicine, Assiut University, Assiut, 71526 Egypt

**Keywords:** Skin healing, Wound, Amniotic membrane, Dogs, Xenogeneic materials, Regenerative medicine, Animal biotechnology, Biomaterials, Regenerative medicine, Tissue engineering

## Abstract

Wound healing is a complex process involving multiple phases aimed at repairing damaged tissues. Disruptions in this process can lead to chronic wounds and infections. Effective treatments that maintain cellular bioactivity while being cost-effective and easy to manufacture and store are needed. The amniotic membrane (AM) is highly biocompatible and rich in bioactive factors, making it valuable for regenerative medicine. Bovine AM is noteworthy for its large size, which facilitates its use in medical settings. However, preserving its bioactivity during storage is a challenge. Therefore, this study aimed to evaluate the effect of bovine lyophilized AM on full-thickness skin wound healing in dogs, compared to that of fresh AM. Bovine AM was collected, lyophilized, and characterized by quantifying growth factors, including vascular endothelial growth factor (VEGF) and basic fibroblast growth factor (bFGF), as well as collagen, glycosaminoglycans (GAGs), elastin, and DNA. Additionally, the surface morphology was imaged using scanning electron microscopy (SEM). The effects of conditioned media from fresh and lyophilized AM on fibroblast and endothelial cell proliferation were compared. In vivo, three full-thickness skin wounds were created on the back in twelve dogs and treated with saline (control), fresh AM, or lyophilized AM, and monitored for healing over 1, 3, and 5 weeks. The fresh AM contained 57.3 ± 6.21 µg/mg collagen, 5.62 ± 1.1 µg/mg GAGs, 11.6 ± 4.52 µg/mg elastin, and 46.3 ± 12.8 ng/mg DNA, with VEGF and bFGF levels of 5.43 ± 2.485 and 1.97 ± 0.482 ng/mg, respectively. The lyophilized AM contained 217.74 ± 8.78 µg/mg collagen, 14.4 ± 1.56 µg/mg GAGs, 43.2 ± 6.8 µg/mg elastin, and 234.6 ± 21.5 ng/mg DNA, with VEGF and bFGF levels of 28.12 ± 7.6 and 13.3 ± 6.89 ng/mg, respectively. SEM revealed a monolayer with poorly defined borders in fresh AM, whereas lyophilized AM displayed a well-defined apical border with few microvilli. Lyophilized AM-conditioned media promoted greater endothelial cell and fibroblast proliferation. Compared with those in the fresh AM and control groups, wounds treated with lyophilized AM healed faster, with narrower edges and more pronounced re-epithelization and collagen remodeling at 1-, 3-, and 5-weeks post-wounding. Histopathology revealed quicker granulation and inflammatory cell infiltration in the first week for lyophilized AM, and better re-epithelization and collagen remodeling in subsequent stages. In conclusion, the amniotic membrane, particularly in its lyophilized form, offers significant benefits for skin wound healing due to its bioactivity, availability, and cost-effectiveness.

**Supplementary Information:**

The online version contains supplementary material available at 10.1038/s41598-025-95023-9.

## Introduction

Skin wounds represent a significant medical and social burden globally^1^. For instance, wounds are estimated to cost the U.S. over $25 billion annually, significantly contributing to the rising cost of healthcare^2^. Additionally, in the veterinary field, skin wounds and their subsequent management pose a significant challenge with financial implications, especially considering that approximately three-quarters of wounds across different animal species heal by secondary intention^3–6^. Cutaneous wounds commonly arise from automobile accidents, burns from heat or fire, surgical damage, contact with sharp objects, animal bites, and gunshot wounds^7^.

Wound healing is a complex and vital process for skin repair in both humans and animals. As a normal biological process, it involves four sequential yet overlapping phases: hemostasis, inflammation, proliferation, and remodeling or resolution^8^. Immediately after a wound occurs, the hemostasis phase begins with vascular constriction and fibrin clot formation to control bleeding. Proinflammatory cytokines and growth factors are released from the forming clot and surrounding area to attract inflammatory cells towards the wound site, initiating chemotaxis. These inflammatory cells infiltrate the wound area to clear invading pathogens and debris^9^. The proliferative phase follows the inflammatory phase, during which the wound surface recovers through re-epithelialization, collagen formation, extracellular matrix (ECM) synthesis, and reconstruction of the vascular network. Finally, the wound healing process enters the remodeling phase, where regenerative processes, including regression of newly formed capillaries and contraction of wound tissue, aim to restore normal architecture^8–10^.

However, certain conditions such as ischemia, pressure necrosis, infection, concurrent systemic diseases, repeated trauma, foreign bodies, and corticosteroid administration can disrupt the standard cascade of wound healing, resulting in delayed or arrested healing processes with associated complications^11–13^. The objective of treating wounds is to enhance the healing process, reduce the required time, and prevent infection and complications^13,14^. To achieve this goal, researchers have developed various products to promote skin healing, including topical agents, dressings, nanomaterials, biological therapies, closure devices, and negative pressure wound therapy^15–24^. Nonetheless, there is still a need for an ideal wound healing product to overcome the limitations of current products.

Amniotic membrane (AM), the inner layer of fetal membranes, has been successfully used as an alternative biomaterial for wound care and reconstructive purposes since its initial application by Davis in 1910 as a surgical material in skin transplantation^25,26^. Although AM has been applied in various clinical scenarios, including wound dressing, ocular surface defects, diabetic foot ulcers, venous ulcers, and various types of postsurgical and post-traumatic wound dehiscence, however, its widespread clinical adoption has been hindered by several limitations^27–32^. These limitations include the difficulty of handling thin AM sheets without folding or tearing, the need for sutures or adhesives to fix the membrane over the wound, and the challenges and costs associated with transportation and storage due to the living characteristics and cellular content of the amniotic membrane^33^. Therefore, this study aimed to create a dried form of an amnion-based wound healing product that is easily storable while preserving the bioactivity and clinical efficacy of the living cellular material.

## Materials and methods

### Ethical statement

All procedures in this study were approved by the Ethical Committee of the Faculty of Veterinary Medicine, Assiut University (Approval Number 06/2023/0109).

The study was conducted in accordance with Egyptian bylaws and OIE animal welfare standards for animal care and use in research and education. All methods were performed in compliance with the Animal Research: Reporting of In Vivo Experiments (ARRIVE) guidelines.

### Isolation of the amniotic membrane

Bovine amniotic membranes were isolated from the placentae obtained from 5 cows undergoing cesarean section at the Obstetrics and Gynecology Department of Assiut University Veterinary Hospital, following uncomplicated pregnancies. All cows exhibited no signs of infection in the placenta/newborn, were delivered on time, and experienced membrane rupture less than 4 h before delivery. Handling and preparation of the placenta were conducted under sterile conditions. The placenta was collected and washed with normal saline immediately after fetal extraction. Subsequently, the amniotic membrane was separated from the other layers and washed 3 times with normal saline containing an antibiotic solution (Vetrocin; El-Nasr Pharmaceutical Chemicals Company, Egypt) for 15 min each. The amnion was then cut into approximately 5 cm × 5 cm pieces using scissors. The amniotic membrane was immersed in fresh normal saline containing an antibiotic, in a sterile collection container, and stored at 4 °C until use. All fresh amniotic membranes were used within 48 h after collection. Sterilization of the amniotic membrane was performed by exposure to ultraviolet rays for 30 min before application.

### Preparation of the amniotic powder

The amniotic membrane was placed into sterile conical tubes and frozen at − 80 °C. The frozen tissue was then placed in a sterilized dish, and liquid nitrogen was added. The amniotic membrane was then pulverized into small fragments. These fragments were transferred to glass flasks and lyophilized using a freeze dryer (VirTis, model #6KBTES-55, Albany, NY, USA). The lyophilized tissue was sterilized by exposure to UV for 30 min and stored in a dry place at room temperature until use.

### Growth factor analysis

Fresh and lyophilized AM (*n* = 5) was suspended in urea-heparin extraction buffer consisting of 2 M urea and 5 mg/ml heparin in 50 mM Tris with protease inhibitors at pH 7.4. The mixture was agitated at 4 °C for 30 h followed by centrifugation at 13,000 × g for 30 min, after which the supernatants were collected. The concentration of total protein in the supernatant was measured by the Bradford protein assay according to the manufacturer’s protocol. Vascular endothelial growth factor (VEGF) and basic fibroblast growth factor (bFGF) were quantified using bovine VEGF and bFGF (MyBioSource) enzyme linked immunosorbent assay (ELISA) kits. The assay was done in triplicate.

### GAGs quantification

GAGs content in the fresh and lyophilized AM was quantified using a dimethyl methylene blue dye-binding assay kit (Blyscan, Biocolor, Carrickfergus County Antrim, UK), following the manufacturer’s instructions. In brief, 5 mg of lyophilized sample (*n* = 5) was homogenized and solubilized. Subsequently, a 100 µL aliquot from each sample lysate was mixed with 1 mL of dimethyl methylene blue and agitated on a shaker at 25 °C for 30 min. Then, the solutions were centrifuged at 10,000 × g for 10 min to collect the GAG-dye complex, and the supernatants were discarded. The resulting pellet was suspended in 1 mL of the provided dissociation reagent, and the absorbance was read at 655 nm.

### Collagen quantification

Total collagen level in the fresh and lyophilized AM was assessed using a Sircol collagen dye-binding assay kit (Biocolor), according to the manufacturer’s instructions. Initially, 5 mg of lyophilized sample (*n* = 5) was homogenized, and total acid pepsin-soluble collagen was obtained after incubation in 0.5 M acetic acid containing 0.1 mg/ml pepsin. A 100 µL aliquot of acid neutralizing reagent was added to the acid-pepsin extract, followed by cold isolation and concentration reagent. After an overnight incubation at 4 °C, the tubes were centrifuged. Then, 1 mL of Sircol dye reagent was added to the pellet and incubated at 25 °C for 30 min. After centrifugation, the pellet was washed with acid-salt wash reagent and suspended in 1 mL of alkaline reagent, and the absorbance was measured at a wavelength of 540 nm.

### Elastin quantification

The elastin content was quantified using a Fastin assay kit (Biocolor) according to the manufacturer’s instructions. Briefly, 5 mg of fresh and lyophilized samples (*n* = 5) were homogenized and incubated in Fastin Dye Reagent. Dye-labeled elastin was then isolated by centrifugation, and the unbound dye was removed. The elastin-bound dye was then eluted and measured spectrophotometrically.

### DNA quantification

DNA was quantified in the fresh and lyophilized AM (*n* = 5) using a QIAGEN DNeasy Blood and Tissue Kit (QIAGEN, Valencia, USA) following the manufacturer’s instructions. Briefly, 5 mg of fresh and lyophilized samples were incubated with cell lysis buffer and proteinase K at 56 °C until complete lysis. The digest was treated with AE buffer and ethanol. DNA was eluted using AE buffer and centrifuged at 8000 rpm for 2 min. The concentration of extracted DNA from each sample was measured with a NanoDrop spectrophotometer ND-1000 (PeqLab, Erlangen, Germany) at an absorbance of 260 nm.

### Scanning electron microscopy

For scanning electron microscopy (SEM), both fresh and lyophilized AM were fixed in a mixture of 2.5% paraformaldehyde and 2.5% glutaraldehyde in phosphate-buffered saline (pH 7.3). The samples underwent a series of steps, including washing in 0.1 M phosphate buffer, dehydration in increasing ethanol grades, critical point drying in liquid carbon dioxide, and gold palladium coating with sputtering equipment. Subsequently, the samples were analyzed and imaged on a camera at the Assiut University EM Center using a JSM-5400LV scanning electron microscope operated at 20 kV.

### Cell proliferation assay

Fresh and lyophilized amniotic membranes (*n*= 5) were incubated in serum-free Dulbecco modified Eagle’s minimal essential medium (DMEM; Invitrogen, Carlsbad, CA, USA) supplemented with 1% penicillin/streptomycin (Pen/Strep, Gibco, Grand Island, NY, USA) at 37 °C/120 rpm for 72 h, following a standard of 0.2 g/mL culture medium^34^. The supernatant was collected and centrifuged to prepare the conditioned media, filtered through 0.4 μm filters, and stored at 4 °C. Human endothelial cell (EC) line EA.hy926 (passage 30; American Type Culture Collection (ATCC)) and mouse embryonic fibroblasts (MEFs) were cultured as monolayers in DMEM supplemented with 10% fetal bovine serum (FBS; HyClone, Logan, UT, USA) and 1% Pen/Strep in a humidified incubator at 37 °C and 5% CO2. Upon reaching 70% confluency, the cells were harvested by trypsinization and used for experiments. Cells were seeded at a density of 5 × 10^3^ in a 96-well plate and incubated for 24 h in complete culture medium. The medium was then aspirated, and 200 µL of conditioned or control medium supplemented with 10% FBS, was added. For the negative control, cells were cultured in complete medium only, while in positive control wells, cells were cultured in the presence of 20% dimethyl sulfoxide (DMSO). Each sample was tested in triplicate. The cellular response to the conditioned media was evaluated after 1, 3, and 7 days of incubation. The metabolic activity of the cells was assessed using [3-(4,5-dimethylthiazol)−2-yl]−2,5- diphenyltetrazolium bromide (MTT) assay. Active cells convert yellow-colored MTT to purple-colored formazan dye crystals. In brief, 20 µL of MTT solution (5 mg/mL; Sigma-Aldrich, St Louis, MO, USA) was added to each well and incubated at 37 °C for 4 h. After removing the medium containing MTT, 200 µL of DMSO was added to all wells to dissolve the formazan to a purple solution. Following a 10-minute incubation, 100 µL aliquots from the wells were transferred to another 96-well plate. The developed color was quantified by measuring the absorbance at a wavelength of 570 nm using a spectrophotometer. Cell viability was expressed as the percentage of activity observed in cells exposed to the conditioned media prepared from fresh or lyophilized AM compared to cells not exposed to the conditioned media (negative control), which was considered as 100%.

### In vivo skin wound induction

This study was conducted on twelve (*n* = 12) clinically healthy mongrel stray dogs of both sexes (8 males and 4 nonpregnant, nonlactating females) weighing between 17 and 30 kg and aged 2–3 years. The dogs were individually housed at the Department of Surgery, Anesthesiology and Radiology, Faculty of Veterinary Medicine, Assiut University with ad libitum access to food and water. Prior to surgery, the dogs were fasted for 12 h. All surgeries were performed under general anesthesia, and all efforts were made to minimize animal suffering and to reduce the number of animals used. Anesthesia was induced with 1 mg/kg xylazine HCL 2% (Xyla-Ject, ADWIA Co., Egypt) and 10 mg/kg ketamine HCl 5% (Ketamine, Sigma-tec Pharmaceutical Industries, Egypt), which were administered intramuscularly in a single syringe. Dogs were positioned on the sternum, and the right side of the midline was prepared for aseptic surgery by clipping, shaving, and disinfecting with alcohol and povidone iodine (Betadine, El-Nile Co. for Pharmaceutical and Chemical Industries, Egypt). The surgical site was draped except for the area where the wound was created.

Three full-thickness excisional skin wounds (3 × 3 cm squares) were created on the right side of the back along the thoracic and lumbar areas of all dogs, with a minimum distance of 4 cm between each wound, resulting in a total of 36 wounds. The 12 dogs were randomly assigned to different time points using an online randomization website (http://www.randomization.com), with 4 animals for each time point. Assignments were concealed in sealed envelopes.

Incisions were made along the skin with a surgical blade to the panniculus carnosus layer, and the overlying skin was excised using a scalpel and scissors. Hemostasis was achieved by applying back pressure with sterile gauze. The cranial wounds were covered with amniotic powder (100 ± 4.2 mg), the middle wounds served as the control, and the caudal wounds were covered with fresh AM (approximately 3 × 3 cm squares, weighing 104 ± 7.8 mg). The amniotic membrane was fixed in the wound using four interrupted stitches with 3/0 Polyglactin 910 (Vicryl, ETHICON, Cincinnati, OHIO, USA). The control wounds were washed with normal saline. All wounds were covered with a sterile cotton pad dressing, gauze, and elastic bandage. The wound dressings were changed weekly. Dogs were administered the antibiotic cefotaxime (Rametax 1 g, Ramida Pharmaceutical and Chemical Industries, Egypt) intramuscularly daily for 5 days after the procedure. Wounds were imaged and subjected to histopathological evaluation at 1-, 3-, and 5-weeks post-wound induction, with 4 dogs evaluated at each time point (*n* = 4 for each time point). Throughout the experiment, the dogs were subjected to clinical observation. Wounds were grossly examined for the presence of abnormal signs such as exudates, infection, or exuberant granulation tissues. No euthanasia of animals was performed during the study.

### Gross evaluation of the wound

Wounds were digitally photographed with a sterile ruler included in each photograph. Wound closure was assessed using ImageJ software (National Institutes of Health, Bethesda, MD, USA), and the results are expressed as a percentage of the initial wound area. This calculation was performed according to the following formula, as previously described:

Percentage Closure = (Initial Wound Area − Current Wound Area/Initial Wound Area) × 100.

### Histological examination

To demonstrate the healing process of skin wounds, samples were obtained at 1, 3, and 5 weeks. Briefly, biopsies were taken to include the wound and surrounding uninjured skin, capturing the full thickness of the skin (epidermis, dermis, and hypodermis) to ensure an accurate representation of the healing process. Biopsies were performed under general anesthesia using 1 mg/kg xylazine HCL 2% and 10 mg/kg ketamine HCl 5%, to avoid any pain to the animals. Following fixation in 10% neutral buffered formalin, the samples were dehydrated using a progressively graded series of ethanol. The samples were prepared according to a previously described protocol^35,36^, involving soaking in methyl benzoate before embedding in paraffin wax (Sigma Aldrich). The sections were then cut at a thickness of 4 μm using a microtome (Richert Leica RM 2125, Germany). These sections were deparaffinized, rehydrated, and processed for routine hematoxylin and eosin (H&E) staining. Following the microscopic examination of the slides, a blind histological evaluation was carried out on coded samples, and a comparison was conducted with the sections from the different treated wounds. The numbers of inflammatory cells (neutrophils, lymphocytes, and macrophages) and newly formed blood vessels infiltrating the granulation tissue in each group were quantified. Using the ImageJ software, five histological fields (400x magnification) per animal in each group were analyzed.

### Collagen histochemical staining

Using Picrosirius red and Crossman’s trichrome, tissue collagen deposition was determined^37^. Under a microscope, the slides were examined for collagen staining, which appeared red with Picrosirius red and green with Crossman’s trichrome.

### Immunohistochemical staining

Immunohistochemical studies were conducted according to a protocol described previously^35,36^. Formalin-fixed, paraffin-embedded tissues were deparaffinized and dehydrated. The sections were then heated for 10 min in sodium citrate buffer (0.01 M, pH 6.0).

After cooling at room temperature for 30 min and washing with PBS, endogenous peroxidase activity was quenched with 3% H2O2 in distilled water for 15 min at room temperature, followed by washing with PBS (2 × 5 min).

The slides were quenched in 3% hydrogen peroxide to neutralize endogenous peroxidase activity, washed in PBS and blocked in 1% bovine serum albumin. The slides were then incubated with a primary antibody against VEGF (rabbit polyclonal antibody, Cat. # RB-222-R7, Thermo Scientific Company) at a dilution of 1:400 overnight at 4 °C. Afterwards, the sections were rinsed three times for 5 min each with PBS and incubated for 15 min with the secondary Ultra Tek HRP Anti-polyvalent antibody (goat anti- rabbit IgG, Scy Tek, TX, USA).

Following these steps, the slides were washed three times for 3 min each with wash buffer. Subsequently, the tissues were incubated with HRP for 15 min, followed by another three washes of 3 min each with wash buffer. The visualization of the reaction was achieved using 3,3’-diaminobenzidine (DAB) diluted with DAB substrate (provided within the same Scy Tek detection kit) according to the manufacturer’s protocol for 10–15 min until the desired staining was visible. The tissues were then counterstained with Harris hematoxylin and mounted with DPX mounting media. All slides were examined using a Leitz Dialux 20 Microscope, and images were captured using a Canon digital camera (Canon Powershot A95).

### Statistical analysis

The data are presented as the means ± standard deviations. IBM SPSS Statistics 20 (SPSS Inc., Chicago, IL, USA) was used for data analysis. An independent t-test was used to analyze the statistical differences between the biochemical components (growth factors, ECM components, and DNA) in fresh and lyophilized amniotic membrane. For comparisons involving the effect of conditioned media prepared from fresh and lyophilized amniotic membrane on cell proliferation, one-way analysis of variance (ANOVA) followed by Tukey’s post hoc test was applied. A *p*-value of < 0.05 was considered statistically significant.

## Results

### Biochemical analysis

**Fig. 1:**
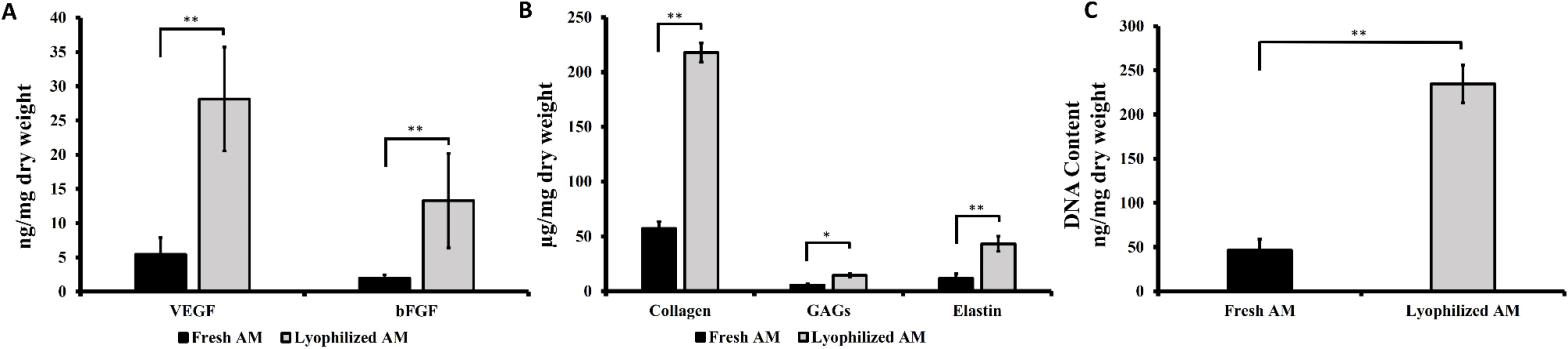
Biochemical characterization of both fresh and lyophilized AM. **A**) Quantification of growth factors, including VEGF and bFGF. **B**) Quantification of collagen, GAGs, and elastin. **C**) Measurement of DNA content in the amniotic membrane. These assays were done in triplicate. Data are presented as mean ± standard deviation, with * indicating significance (*p* < 0.05) and ** indicating highly significant differences (*p* < 0.01).

To assess whether the amniotic membrane contains essential growth factors for initiating the wound healing process, the levels of VEGF and bFGF were quantified. The lyophilized amniotic membrane (AM) showed significantly higher levels of VEGF (28.12 ± 7.6 ng/mg) and bFGF (13.3 ± 6.88 ng/mg) compared to the fresh AM (5.43 ± 2.41 ng/mg and 1.97 ± 0.48 ng/mg, respectively; *p* < 0.01) (Fig. 1A).

The levels of extracellular matrix (ECM) components, including glycosaminoglycans (GAGs), collagen, and elastin, were also quantified. Lyophilized AM exhibited significantly higher amounts of GAGs (14.4 ± 1.56 µg/mg), collagen (217.74 ± 8.78 µg/mg), and elastin (43.2 ± 6.8 µg/mg) compared to the fresh AM (5.62 ± 1.1 µg/mg, 57.3 ± 6.21 µg/mg, and 11.6 ± 4.52 µg/mg, respectively; *p* < 0.05 for GAGs and *p* < 0.01 for collagen and elastin) (Fig. 1B).

Additionally, the DNA content was significantly higher in lyophilized AM (234.6 ± 21.5 ng/mg) compared to fresh AM (46.3 ± 12.8 ng/mg; *p* < 0.01) (Fig. 1C).

### Scanning electron microscopy

SEM analysis of the fresh AM revealed a monolayer with less distinct borders and a surface coated with microvilli and secretions on the apical side of the cells. Collagenous fiber bundles were observed in the stroma of the fresh AM (Supplementary Fig. 1). On the other hand, SEM of lyophilized AM revealed a well-defined apical border with few microvilli and no apical secretion. The network of multidirectional reticular fibers that made up the stroma of the lyophilized AM is shown in Supplementary Fig. 2.

### Cell proliferation assay

**Fig. 2:**
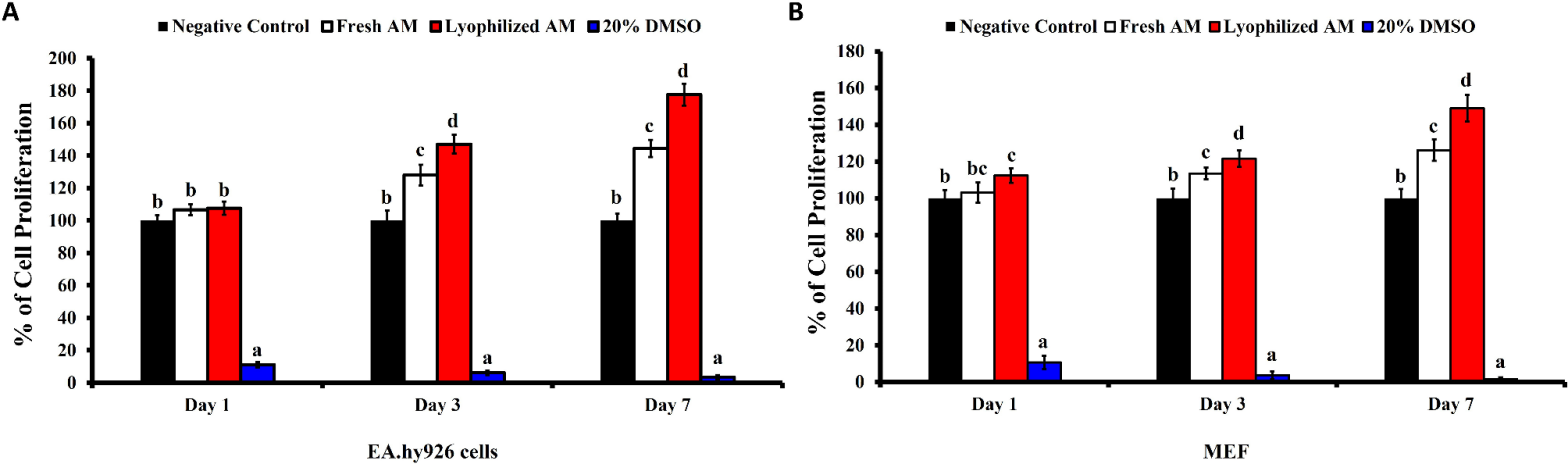
Effect of conditioned media prepared from fresh AM and lyophilized AM on cell proliferation. **A**) Evaluation of EA.hy926 endothelial cells and **B**) MEF proliferation at days 1, 3, and 7 after incubation with conditioned media. The assay was done in triplicate. The data are presented as the mean ± standard deviation. The negative (basal media) control is considered as 100%. Statistical analysis was performed using one-way ANOVA followed by Tukey’s test. Groups labeled with different letters (e.g., ‘a’, ‘b’, ‘c’) are statistically significantly different from each other (*p* < 0.05), while those sharing the same letter are not.

As shown in Fig. 2A, there was no significant difference in the proliferation rate of EA.hy926 endothelial cells after 1 day of culture. However, compared with the control, both fresh and lyophilized AM resulted in significantly greater proliferation after 3 and 7 days of incubation. Compared with the negative control media and the conditioned media prepared from fresh AM (127.9 ± 6.39% and 144.38 ± 5.32%, respectively), lyophilized AM significantly increased the proliferation rate by 147.04 ± 5.8% and 177.58 ± 6.74%, respectively, after 3 days of culture.

Regarding the effect of conditioned media on fibroblasts, the lyophilized AM showed a significantly higher proliferation rate (112.3 ± 3.89%) of MEFs compared to the control, with no significant difference from the fresh AM (103.1 ± 5.57%) after 1 day of incubation (Fig. 2B). The lyophilized AM demonstrated a significantly higher proliferation rate (121.6 ± 4.62% and 148.9 ± 7.25) compared to fresh AM (113.5 ± 3.1% and 126.2 ± 5.95%) and the negative control, after 3 and 7 days of incubation, respectively.

### Fresh and lyophilized amniotic membranes accelerate wound healing in vivo

**Fig. 3:**
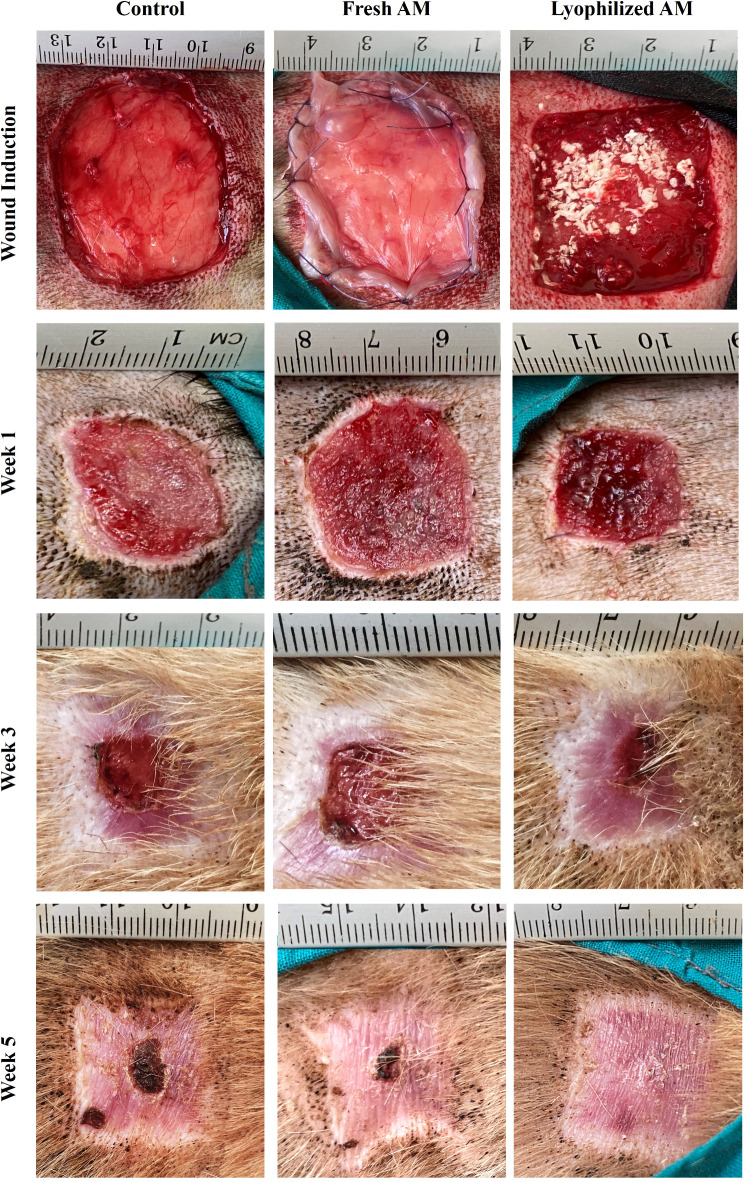
Gross evaluation of the skin wounds in dogs (*n* = 12; 4 for each time point). Representative photographs of the skin wound area of the control, fresh AM, or lyophilized AM groups after 1, 3, and 5 weeks of wound induction. The ruler units are mm.

**Fig. 4:**
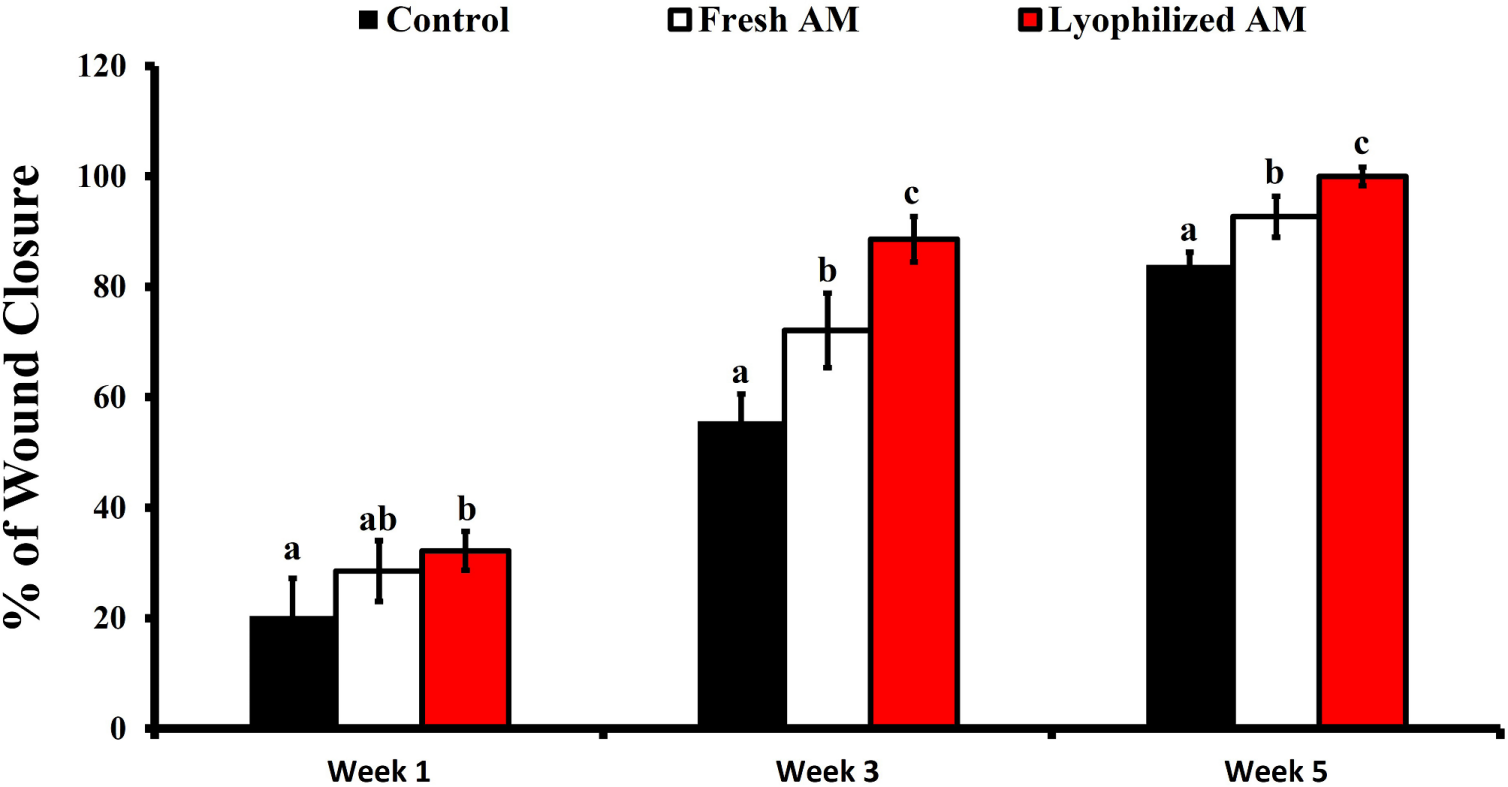
Measurement of the wound closure percentage using ImageJ. The results were compared to the initial wound area. The data are presented as the mean ± standard deviation. Statistical analysis was performed using one-way ANOVA followed by Tukey’s test. Groups labeled with different letters (e.g., ‘a’, ‘b’, ‘c’) are statistically significantly different from each other (*p* < 0.05), while those sharing the same letter are not.

To investigate the effect of fresh and lyophilized AM on wound healing, skin wounds were treated with fresh or lyophilized AM, or saline. Complete wound closure was observed over 5 weeks, and the wounds were photographed at 1-, 3-, and 5-weeks post-wound induction. The results showed that lyophilized AM-treated wounds had narrower edges, and the fastest healing rate compared to fresh amniotic membrane and the saline-treated group over the treatment period, at 1-, 3-, and 5-weeks post-wounding (Fig. 3). The wound closure rate was determined according to the percentage of the initial wound area. At week 1, the lyophilized AM group showed significant wound closure rate (32.2 ± 3.5%) compared to the control group (20.4 ± 6.8%), with no significant difference compared to the fresh AM group (28.5 ± 5.5%) as shown in Fig. 4. At weeks 3 and 5, the wounds treated with lyophilized AM exhibited significantly higher closure percentages (88.6 ± 4.1 and 100 ± 1.7%, respectively) than did those in the control (55.7 ± 4.9% and 84 ± 2.29%, respectively) and fresh AM (72.1 ± 6.72% and 92.7 ± 3.7%, respectively) groups.

### Histological evaluation of wound healing for re-epithelization and neovascularization

**Fig. 5:**
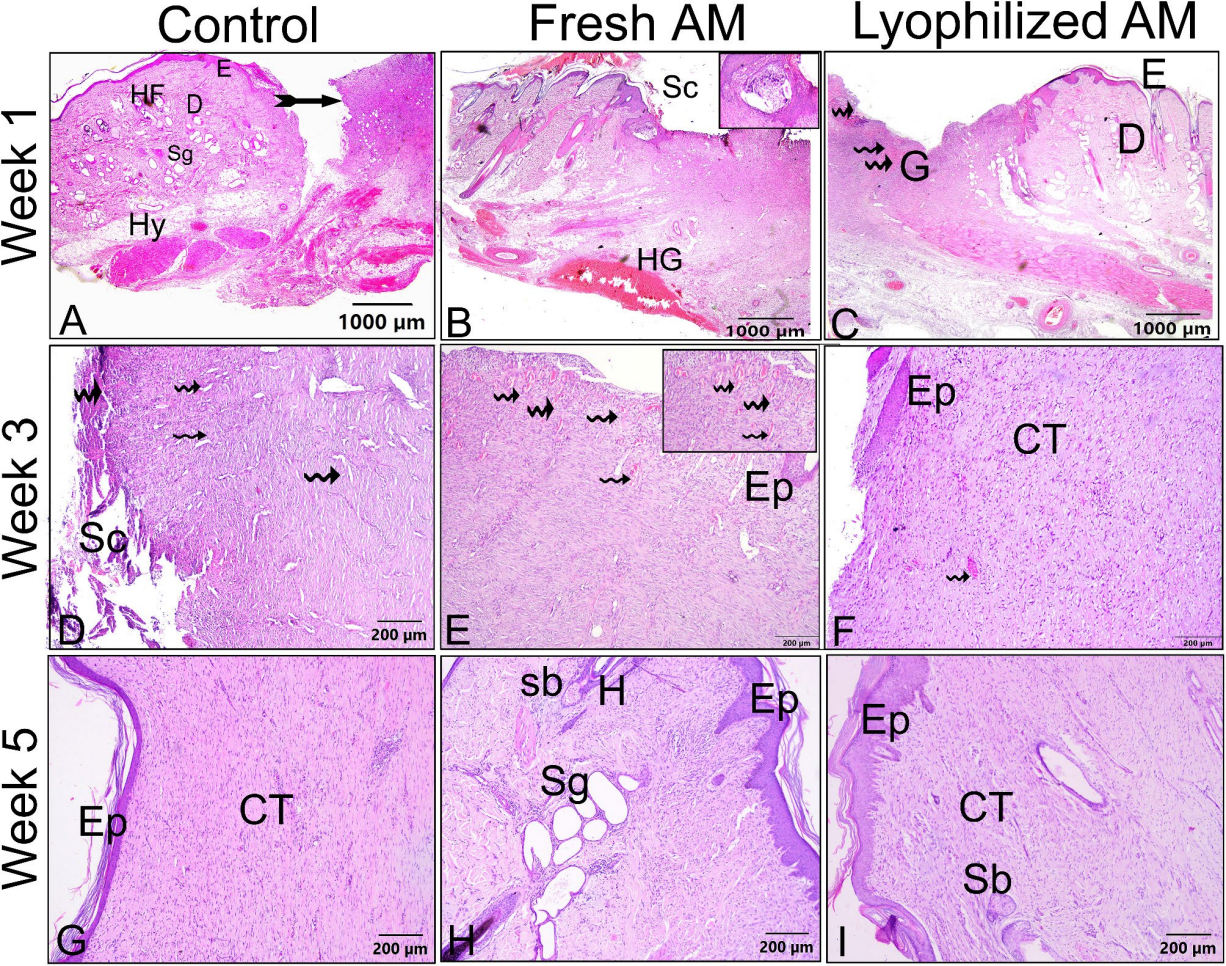
H&E staining of skin wounds from the different groups. **A–C** The wound region one week after wound induction. **A** Control group showing an opened wound area (forked arrow). **B** The fresh AM group showing granulation tissue with an influx of inflammatory cells (boxed inset). **C** Lyophilized AM group with pronounced granulation tissue (**G**) and angiogenesis (wavy arrows). **D-F** The wound area in the different groups 3 weeks after wound induction. **A** Control groups showing scabs (Sc) and blood vessels in the wound area (wavy arrows). **B** Fresh AM showing many blood vessels in the wound area (wavy arrows and inset box). Re-epithelization could be detected in fresh AM (Ep). **C** The lyophilized AM group showed re-epithelization of the epithelium (Ep) and a few new blood vessels (wavy arrow). **G-I** The wound area in the different groups 5 weeks after wound induction. The skin was relatively normal in all groups, consisting of epidermis (**E**) and dermis with remodeled connective tissue (CT). However, skin appendages were absent in the control group, and they were more abundant in the fresh AM and lyophilized AM groups. Abbreviations: **E**: epidermis; **D**: dermis; **Hy**: hypodermis; **HG**: hemorrhage; **Sb**: sebaceous gland; **H**: hair follicle; **Sg**: sweat gland.

**Fig. 6:**
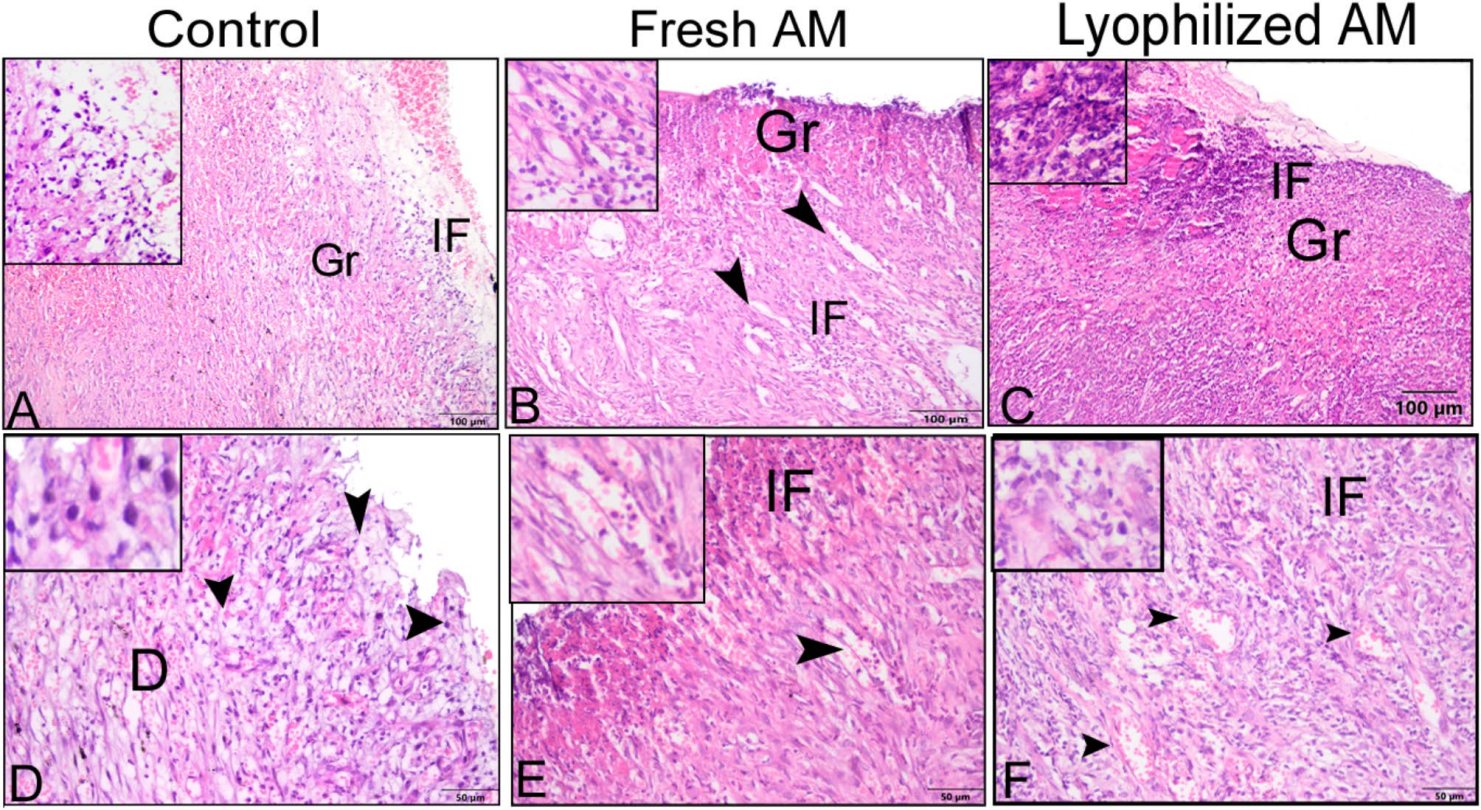
Paraffin sections stained with H&E showing the histological composition of skin wounds 1 week after wound induction. In the control (**A**,** D**) and the fresh AM (**B**,** E**) groups, new blood vessels formed in the dermal layers (inset), whereas more superficial new blood vessels (arrowheads) formed in the granulation tissue (Gr) in the lyophilized AM group. The lyophilized AM group (**C**,** F**) exhibited more prominent inflammatory cell infiltration (IF, an inset). Abbreviation: E: epidermis.

**Fig. 7:**
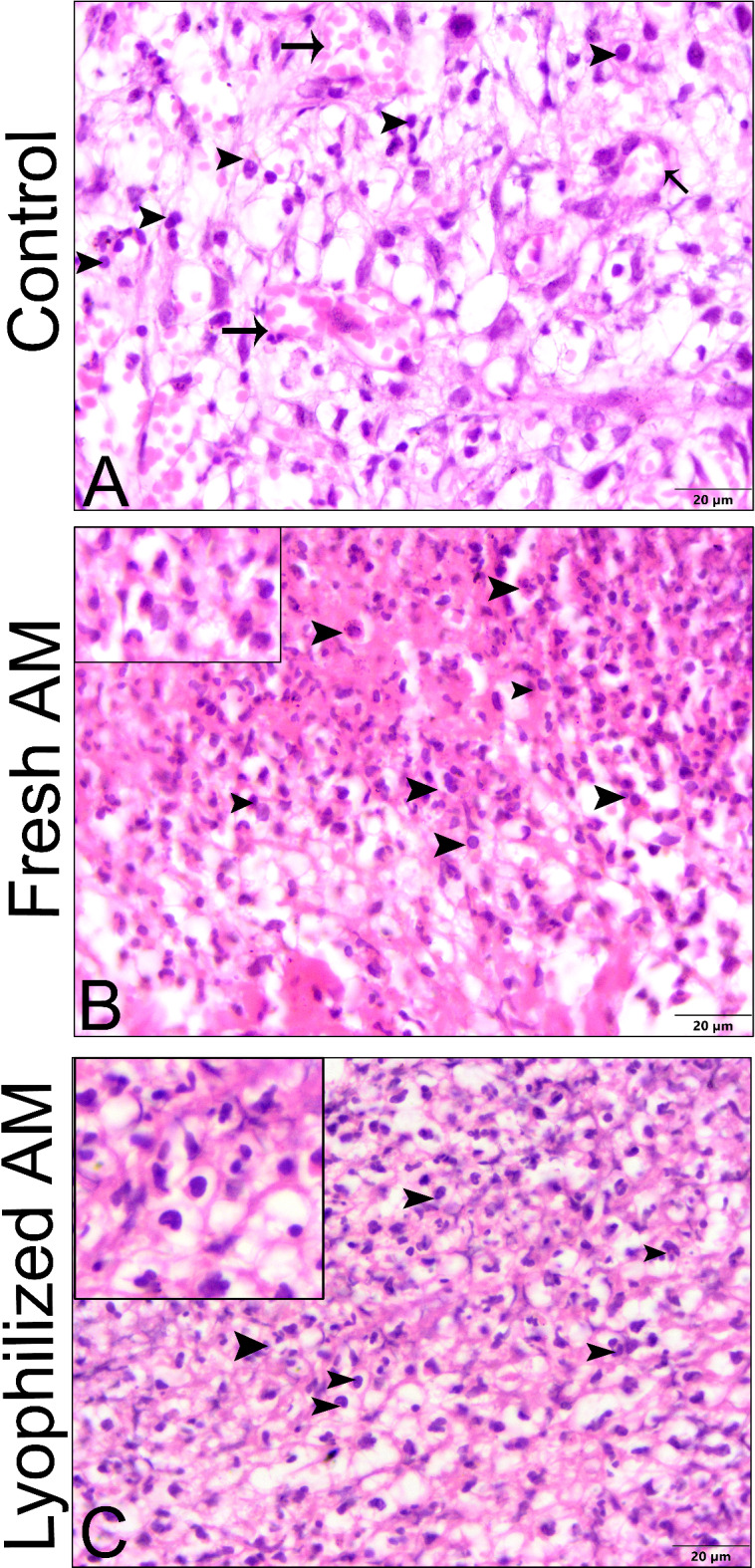
Higher magnification images of skin wound after 1 week. (**A–C**) Compared to control groups, both the fresh AM and lyophilized AM powder groups showed a more pronounced and extensive leukocyte infiltration (arrowheads and insets) in the granulation tissue (Fig. 7). A blood vessel is indicated by the arrow, and fibroblast cells are marked by a wavy arrow.

**Fig. 8:**
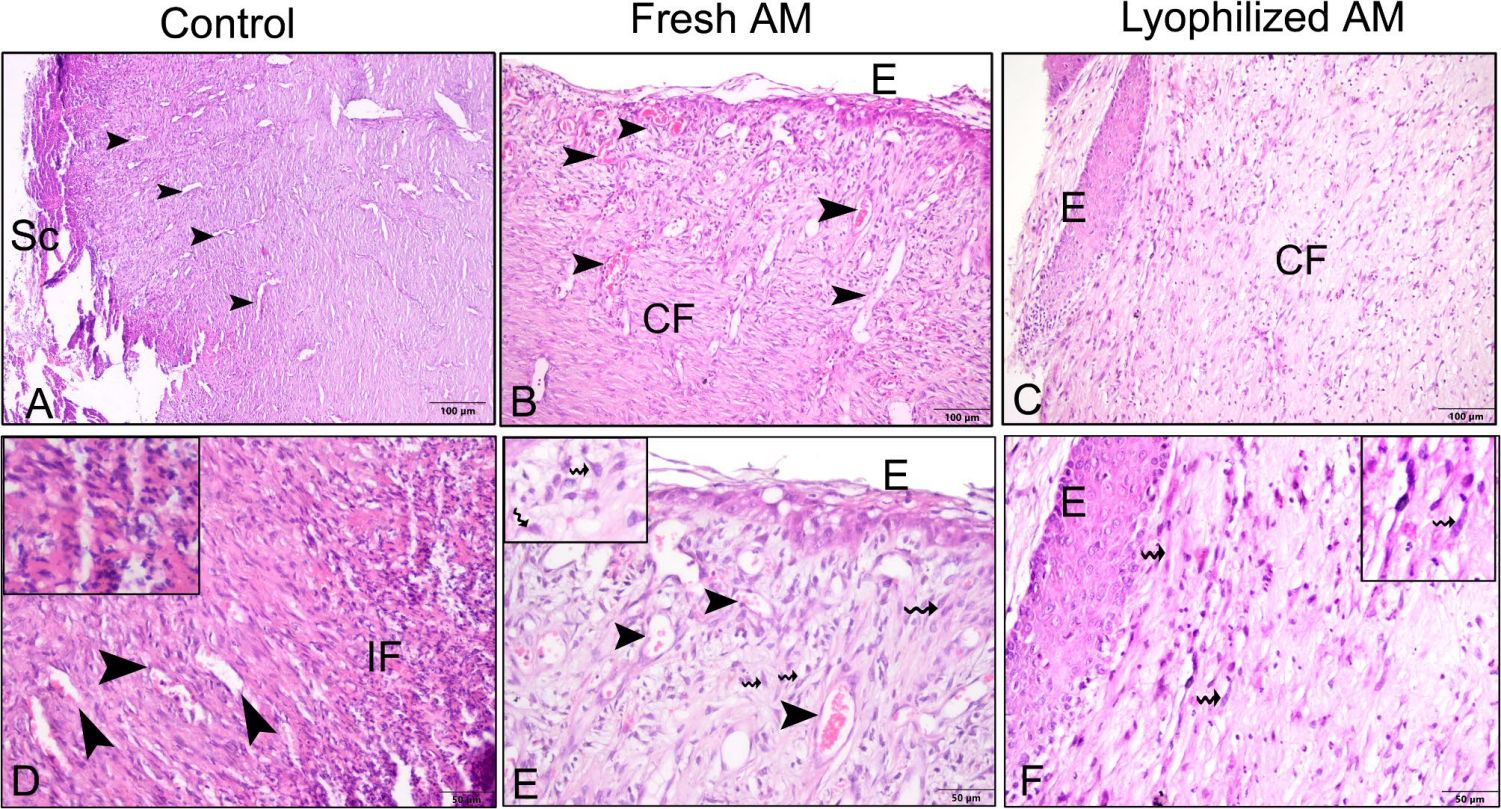
Paraffin sections stained with H&E showing the histological composition of skin wounds 3 weeks after wound induction. The control group (**A**,** D**) showed scabs (Sc) intermingled with inflammatory cells (IF, arrowheads). (**B**,** E**) The fresh AM group exhibited re-epithelialization (**E**), and numerous blood vessels (arrowheads). (**C**,** F**) The lyophilized AM group also showed re-epithelization (**E**). (**E**,** F**) Wavy arrows and insets highlight prominent fibroblast cell proliferation and collagen fiber deposition (**CF**).

The histological sections were subjected to a descriptive microscopic examination to evaluate the wound healing process. Wounds treated with normal saline were utilized as negative controls for comparison with wounds treated with fresh AM and lyophilized AM powder. The structure of normal skin comprises the epidermis, dermis, and hypodermis. The epidermis consists of thin, stratified, keratinized squamous epithelium, which invaginates into the dermis to form hair follicles and associated sebaceous glands (Fig. 5A-C). One week after wound induction, the groups treated with fresh AM and lyophilized AM powder exhibited markedly greater wound closure compared to the negative control group (Fig. 5A-C). All groups showed signs of epidermal necrosis (total loss of the epidermis), leukocyte infiltration, and severe dermal hemorrhage (Fig. 6A-F). Granulation tissue filled the base of the wound, characterized by the infiltration of inflammatory cells within a loose extracellular matrix, along with the proliferation of fibroblasts and the formation of thin-walled, delicate capillaries (angiogenesis) (Figs. 6 and 7). In comparison to control groups, the fresh AM group and lyophilized AM powder showed a more noticeable and extensive infiltration of leukocytes, including neutrophils and monocytes, into the granulation tissue (Fig. 7A-C, supplementary Fig. 3.). Furthermore, the lyophilized AM group had a considerably higher number of newly formed blood vessels than the control and fresh AM groups (Supplementary Fig. 3). Following 3 weeks of wound induction, the control group showed no signs of re-epithelialization and still had scabs intermingled with inflammatory cells (Figs. 5D and 8A and D). In contrast, wounds treated with fresh AM and lyophilized AM exhibited more extensive re-epithelialization (Figs. 5E and F and 8B and C). Upon comparing the lyophilized AM group to the fresh AM and control groups, less granulation tissue, reduced leukocyte infiltration, and angiogenesis were observed (Fig. 8B-F, Supplementary Fig. 4). Nonetheless, fibroblast proliferation was observed in the lyophilized AM and fresh AM groups, together with increased formation of new connective tissue (Fig. 8C, F). Five weeks after wound induction, the skin of the wounds treated with lyophilized and fresh AM was remarkably similar to that of normal skin, exhibiting no scarring, a thin epidermis, remodeled connective tissue, and nearly normal proliferation of hair follicles and sebaceous glands on the wound surface (Fig. 5H, I). In contrast, the control wounds had normal skin but no discernible sebaceous glands or associated hair follicles (Fig. 5G).

**Fig. 9:**
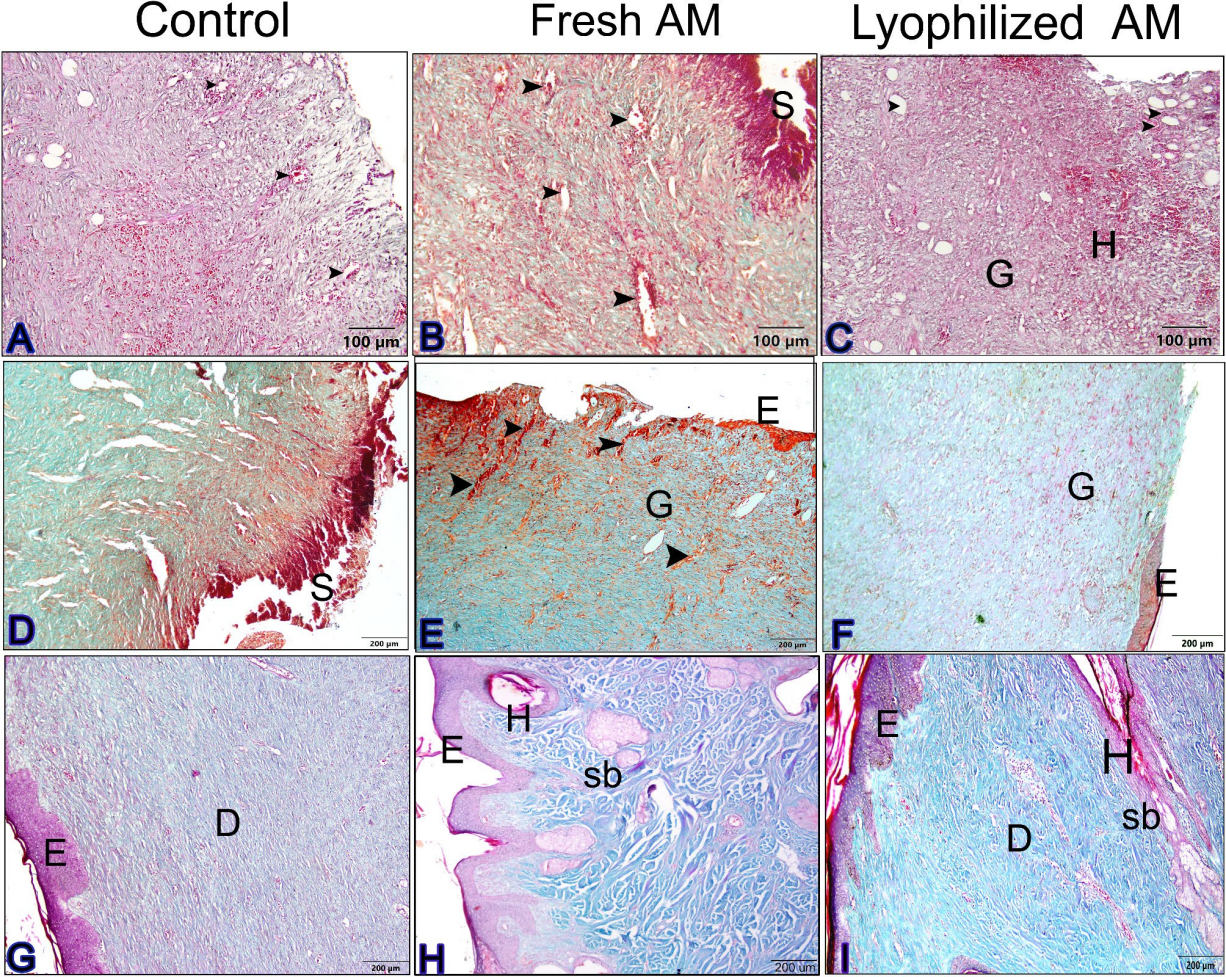
Histological composition of skin wounds among different groups stained with Crossman’s trichrome. **A-C** The wound region 1 week after wound induction. Collagen was stained green using Crossman’s trichrome, while red blood cells, muscle, and cytoplasm were stained red. One week after wound induction, the granulations (**G**) in each group showed reddish staining together with sporadic green deposits of collagen. Arrowheads point to newly formed blood vessels. **D-F** The wound area 3 weeks after wound induction. Compared to those in the control (**D**) and fresh AM groups (**E**), collagen deposits (green) were more abundant in the lyophilized AM group (**F**). **G-I **The wound area 5 weeks after wound induction. Compared to those in the control (**G**) and fresh AM (**H**) groups, the wounds that received lyophilized AM powder (**I**) had the greatest amount of remodeled collagen fibers. Insets show angiogenesis in the wound areas. Abbreviations: S: scab; Sb: sebaceous gland; H: hair follicle; E: epidermis; D: dermis.

**Fig. 10:**
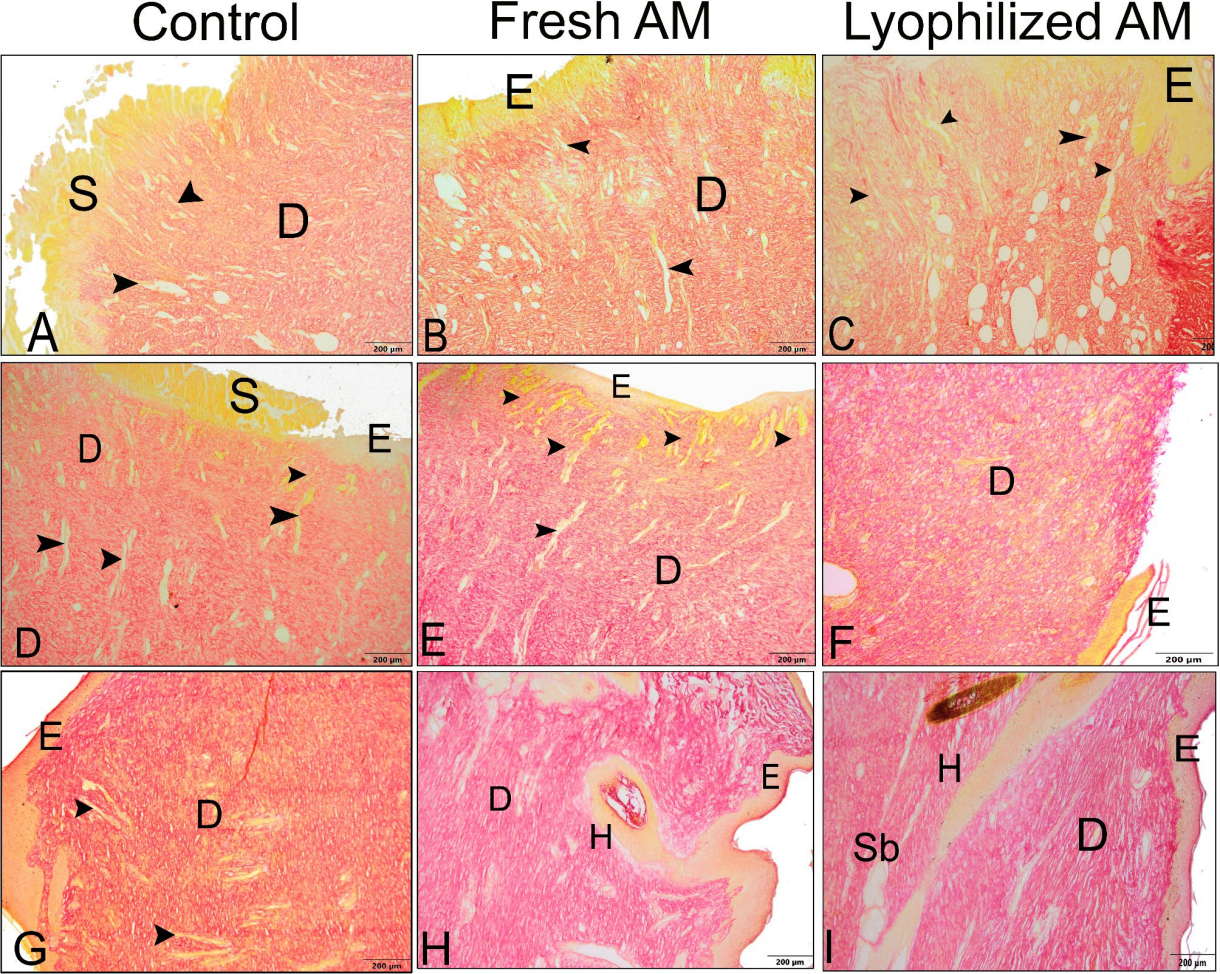
Histological composition of skin wounds stained with Picrosirius red in the different groups. **A-C** The wound region one week after wound induction. Collagen was stained red using Picrosirius red, while red blood cells, muscle, and cytoplasm were stained yellow. **D-F** The wound area 3 weeks after wound induction. **G-I** The wound area after 5 weeks from the wound induction. Picrosirius red staining revealed that the wounds in the lyophilized AM treatment group exhibited a quicker healing process in terms of neovascularization (arrowheads), re-epithelialization (**E**), early keratinocyte formation, and related adnexa. Abbreviations: S: scab; Sb: sebaceous gland; H: hair follicle; Sg: sweat gland; D: dermis.

Collagen synthesis by fibroblasts plays a crucial role in wound healing. To evaluate collagen deposition during the process of matrix remodeling and granulation tissue development, paraffin sections were stained with Picrosirius red and Crossman’s trichrome. Collagen was stained green with Crossman’s trichrome, while epithelial cells, muscles, and cytoplasm were stained red (Fig. 9). Collagen types can be distinguished by their colors using Crossman’s trichrome staining: Type I collagen appears reddish-orange, while Type III collagen is yellowish green. One week after wound induction, the granulations in each group showed reddish staining together with sporadic green deposits of collagen (Fig. 9A-C). The lyophilized AM group exhibited greenish-colored wounds with increasing collagen deposition as the wounds became older (Fig. 9D-F). Compared with those in the control and fresh AM groups, the wounds that received lyophilized AM powder had more collagen fibers (green color) 5 weeks after wound induction. Similar findings were observed with Picrosirius red staining, which stains collagen red while staining muscle, red blood cells, sebaceous glands, and epithelium yellow (Fig. 10). Picrosirius red staining revealed that the lyophilized AM-treated group exhibited a quicker healing process in terms of neovascularization, re-epithelialization, and early keratinocyte formation.

**Fig. 11:**
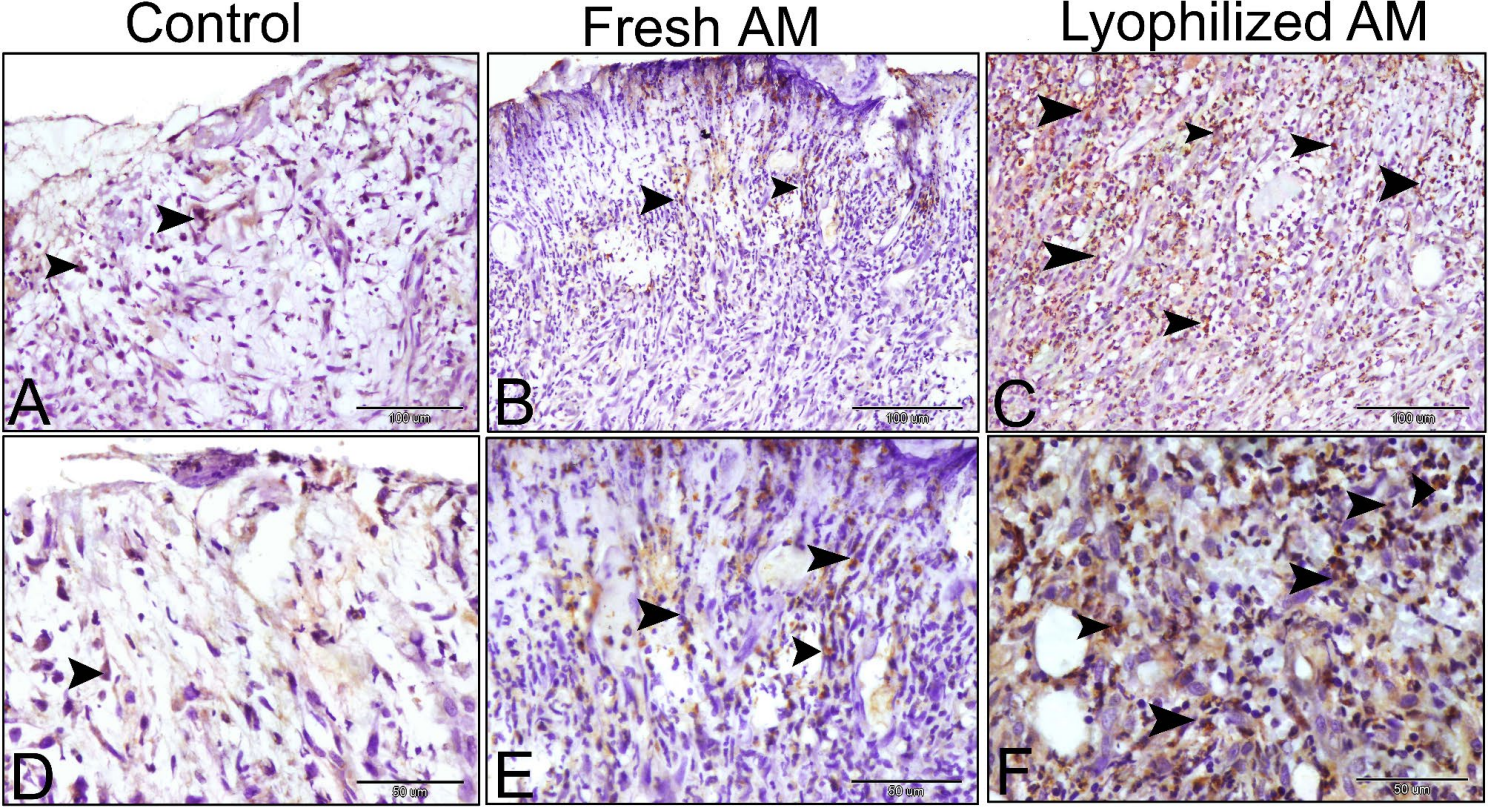
VEGF immunohistochemical expression in the wound tissue 1 week after wound induction. VEGF expression was more pronounced in the granulation tissue of the lyophilized AM group (**C**,** F**) than in that of the control (**A**,** D**) and fresh AM groups (**B**,** E**) at week 1 post-wounding. It was noted in the endothelial cells of newly created microvessels and in inflammatory and fibroblasts (arrowheads).

**Fig. 12:**
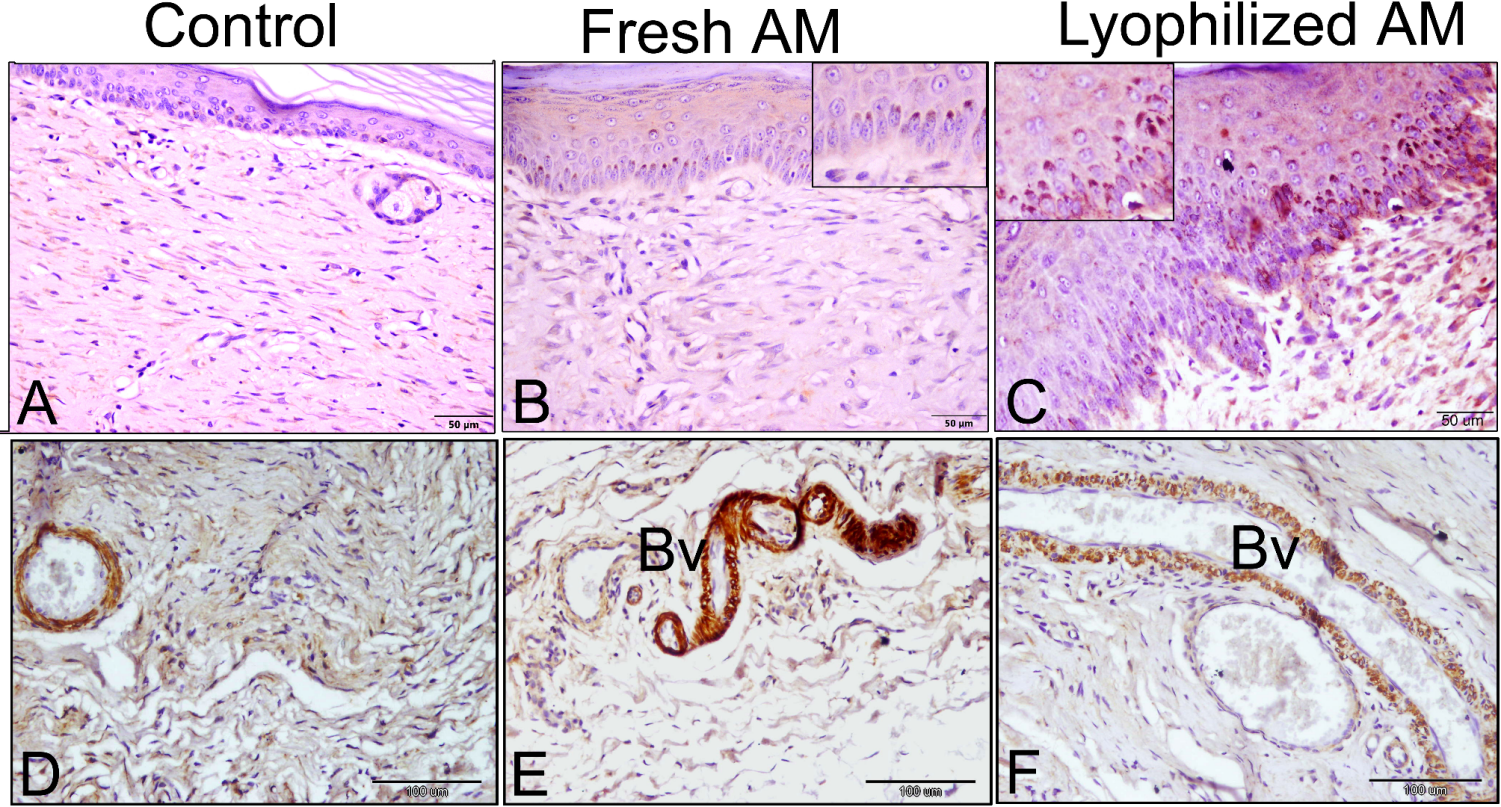
VEGF immunohistochemical expression in the wound tissue 5 weeks after wound induction. **A**) VEGF expression in the dermal and epidermis was weak in the control groups. **B**) VEGF expression in Fresh AM groups was primarily limited to the keratinocyte basal layer of the epidermis (arrowhead; inset in **B**). **C**) In the lyophilized AM group, the expression was detected in the sebaceous glands (Sb) and the internal and external root sheaths of hair follicles (arrows). **D-F**) VEGF expressed in the dermal regions of the different groups’ blood vessels (Bv).

Angiogenesis, an essential mechanism for maintaining granulation tissue and accelerating wound healing, is induced by various angiogenic agents, including VEGF. VEGF expression was more pronounced in the granulation tissue of the lyophilized AM group than in those of the control and fresh AM groups at 1-week post-wound. VEGF was expressed in the microvessels of the granulation tissue, as well as in activated fibroblasts and inflammatory cells, including macrophages, during week 1 following wound induction (Fig. 11). By week 5, VEGF expression was primarily confined to the keratinocyte basal layer of the epidermis, particularly in the fresh and lyophilized AM-treated groups. Additionally, VEGF was expressed in the tunica media of blood vessels within the dermal layer (Fig. 12).

## Discussion

This study illustrates the effectiveness of both fresh AM and lyophilized AM powder in treating full thickness wounds and facilitating rapid wound closure, primarily through enhanced epithelialization. Histological analysis supported these observations, indicating accelerated healing and the formation of a mature epidermis and dermis comparable to those of healthy skin. Both fresh and lyophilized AM exhibit superior wound closure and epithelialization, as well as reduced contraction, suggesting enhanced healing and healthier epidermal coverage.

The repair of skin lesions is considered one of the most intricate biological processes in human physiology and is orchestrated by a cascade of overlapping biochemical and cellular events^9,13^. To effectively stimulate the regeneration process and prevent wound healing failure, a variety of traditional therapies and natural products have been employed, often yielding promising results^2,18^. One therapeutic alternative that has gained significant attention is the utilization of AM derived from the placenta due to its high abundance in stem cells^26^. In comparison to other tissues, the placenta exhibits low immunogenicity and high regenerative properties, and is rich of growth factors, collagens, and GAGs^38–40^.

Collagen plays a fundamental role in regulating structural properties and promotes cell migration, angiogenesis, and granulation tissue formation. It is crucial for new tissue formation and microvessel sprouting and anastomosis after wounding. Additionally, collagen can generate fibers with enhanced strength and stability through self-aggregation and cross-linking. Consequently, collagen has been used as a scaffold for enhancing wound healing in several studies^41,42^.

GAGs play a crucial role in ECM assembly, organization, and tissue repair processes^43^. The amniotic membrane, which is rich in GAGs, enhances wound healing by promoting angiogenesis and inhibiting inflammation^44^. Moreover, sulfated GAGs within ECM hydrogels bind to growth factors and cytokines, prolonging their half-life and enhancing therapeutic effects^45,46^. GAGs and GAG-based biomaterials have been extensively investigated for their potential to facilitate in situ tissue regeneration and repair by modulating the wound microenvironment, hastening re-epithelialization, and managing ECM remodeling. GAGs serve as mediators of biological signals that stimulate cell growth, migration, differentiation, and apoptosis, thereby influencing neo-tissue organization^43^. Given their multifaceted roles in maintaining internal environment homeostasis, exceptional biocompatibility, inherent biodegradability, and adaptable modifiability, GAGs have emerged as ideal candidates for advanced biomaterials aimed at enhancing wound healing^43,47^.

This makes AM an excellent scaffold for tissue integration, providing an optimal environment for cell growth and differentiation. The amniotic membrane releases essential cytokines conducive to wound healing, including platelet-derived growth factor, VEGF, angiogenin, TGF-β2, TIMP-1, and TIMP-2 ^38^. Since the early 20th century, the amniotic membrane has been utilized for various therapeutic purposes, including skin transplantation, urinary bladder reconstruction, ocular lesion treatment, burn care, varicose ulcer management, neovagina reconstruction, and nerve damage repair^48,49^.

In this study, the amniotic membrane showed the presence of growth factors, including VEGF and bFGF, that are important for angiogenesis and fibroblast proliferation and migration. These growth factors can be released from the amniotic membrane leading to enhanced fibroblast and endothelial cell proliferation, as confirmed by the use of conditioned media from fresh or lyophilized amniotic membranes. Corral et al. investigated the importance of VEGF in healing skin ulcers in normal and ischemic ears in rabbits and reported that VEGF seems to be more important than bFGF during ischemic wound healing^50^. Akita et al. investigated the effect of amniotic fluid from humans during mid-gestational trimester on cell properties. They used amniotic fluid to enhance both adult and fetal fibroblast mitogenic activities, including DNA synthesis and cell proliferation^51^. Preincubation with both a bFGF receptor blocker and an anti-bFGF antibody significantly reduced proliferative activity in both adult and fetal skin fibroblasts. Additionally, in the treatment of deep burns in a patient using drainage-slit artificial dermis with bFGF spraying, the majority of the skin healed with a spotted well-vascularized raw surface, indicating that bFGF may enhance wound healing by normalizing the tissue texture and color to match the surrounding intact skin and optimally promoting the healing process.

Both bFGF and VEGF stimulate various cells, including dermal fibroblasts, keratinocytes, endothelial cells, and melanocytes, promoting tissue remodeling, wound healing, and neovascularization due to their mitogenic and angiogenic properties^52,53^. Despite several advantages, such as their multifunctional role in stimulating cell growth and tissue repair, bFGF and VEGF have very short biological half-life when injected and are unstable in solution. Additionally, rapid enzymatic degradation makes it challenging to apply them in their free form, thereby preventing the attainment of effective concentrations for wound healing treatment^54^. Thus, the use of the fresh amniotic membrane can provide sustained release of bFGF and VEGF; additionally, lyophilized amniotic powder can provide the wound with a concentrated amount of these growth factors. This may explain the higher cell proliferation observed with using conditioned media prepared from the lyophilized AM, likely due to the increased concentration of bioactive components per mg resulting from the reduced water content in the lyophilized state.

Moreover, the amniotic membrane exhibits antibacterial capabilities, secreting antimicrobial peptides that augment wound closure and prevent infections^44^. Cryopreserved amniotic membrane has demonstrated efficacy in promoting wound closure and preventing wound-related infections, indicating its potential in clinical applications^55^.

Our quantitative DNA analysis demonstrated that the lyophilized amniotic membrane contained 234.6 ± 21.5 ng/mg DNA. This concentration is notably higher than the 50 ng/mg threshold suggested by various studies to prevent exacerbation of the inflammatory response post-implantation^56,57^. However, recent research by Record Ritchie et al. provided another perspective^58^. They injected 50 µg of DNA, isolated from unprocessed porcine small intestine ECM and decellularized ECM, into mice, with or without potent co-stimulators such as incomplete Freund’s adjuvant, methylated bovine serum albumin, and interleukin-12. Surprisingly, no rejection response was triggered by DNA from the porcine ECM. Instead, a localized mild accommodation cytokine response occurred, with no systemic anti-DNA antibody expression, even at doses significantly higher than those expected from ECM implantation. This suggests that higher DNA concentrations in the amniotic membrane may not necessarily lead to adverse inflammatory responses, highlighting the complexity of immune reactions to implanted biomaterials^59^.

Although the use of amniotic membrane in clinical settings offers clear advantages, its integration into routine clinical practice has been challenging. Limitations include the difficulty of handling thin amniotic membrane sheets without folding or tearing, as well as the need for sutures or adhesives to secure the membrane over the wound. Furthermore, similar to current tissue-engineered skin substitutes, the transportation and storage of living cellular tissue introduce complexity and costs for routine clinical applications^33^. Therefore, our objective has been to develop an amnion-based wound-healing product that does not rely on a living cellular component but still maintains the bioactivity and clinical efficacy of a living cellular product.

Over the years, the preservation of the amniotic membrane has undergone significant development, benefiting from advancements in technology, preparation methods, and sterilization procedures. In 1940, Chao et al. recommended drying human AM before use to alleviate the discomfort associated with fresh AM^60^. The introduction of dry human AM marked a milestone in tissue reconstruction as it proved to be safe and addressed the limitations of using fresh human AM. Subsequently, Kim et al. proposed cryopreservation of human AM by storage at −80°C using a storage medium composed of glycerol and Dulbecco’s modified Eagle Medium (DMEM)^61^. However, cryopreservation alters the composition and distribution of the ECM, resulting in poor cell viability in the amniotic membrane. Moreover, cryopreservation reduces the production of growth factors and cytokines by the amniotic membrane, impacting its structural and mechanical properties^62^. In 2004, Nakamura et al. introduced freeze-dried human AM as a novel preservation method for use as a substrate in ocular surface reconstruction, demonstrating its feasibility^63^. However, both cryopreservation and lyophilization have drawbacks because they may influence amniotic membrane characteristics. In our study, we have proven the retention of the bioactivity of the amniotic membrane after freeze-drying, which suggests the possibility of using freeze-drying for the storage of the amniotic membrane before application.

Branski et al. conducted a comparative study on the application of the amniotic membrane for children with partial-thickness facial burns compared to standard topical treatment^64^. Participants were divided into two groups, with one receiving an amniotic membrane and the other receiving topical antimicrobial. The findings revealed that the use of amniotic membrane for wound coverage was both safe and advantageous compared to the use of standard topical ointments.

In the current study, the application of lyophilized AM showed a better healing response compared to fresh AM. This improved healing response may be attributed to several factors. Firstly, the lyophilization process reduces the water content of the amniotic membrane, thereby concentrating bioactive components such as growth factors, cytokines, and extracellular matrix proteins. This increased concentration can enhance the therapeutic efficacy of the AM. Although the amounts of fresh and lyophilized AM used in vivo were nearly similar in weight (104 ± 7.8 mg for fresh AM and 100 ± 4.2 mg for lyophilized AM powder), fresh AM contains a significant proportion of water, whereas lyophilized AM consists primarily of dry matter. Consequently, the concentration of biologically active components, including essential growth factors such as bFGF and VEGF, is higher per unit weight in the lyophilized AM group. This likely explains the enhanced cellular proliferation observed in vitro and the improved wound healing outcomes in vivo. Secondly, lyophilized AM may offer greater stability and a more controlled release of bioactive factors, which can contribute to improved wound healing. Additionally, the lyophilization process can result in a more stable matrix that supports better cell attachment and proliferation.

These findings are supported by histopathological data, which indicate that in the lyophilized AM group, the proliferative phase of granulation tissue and the influx of inflammatory cells occurred more quickly and distinctly during the first week following wound creation. Additionally, compared to the other groups, the lyophilized AM group showed more noticeable re-epithelization, fibroblast proliferation and remodeling of collagen fibers at 3 and 5 weeks after wound induction. Fibroblasts are the predominant cell type in the wound, and their proliferative phase, which begins approximately 4 days after injury, may last up to 2 or 3 weeks^65^. At the wound site, fibroblasts are stimulated and develop into myofibroblasts, which exert contractile forces that pull the wound edges together and aid in wound closure^66^. Fibroblasts are dynamic and essential cells in wound healing. They multiply and secrete pro-angiogenic chemicals, such as VEGF, and angiopoietin 1 (Ang-1)^67^. These molecules support angiogenesis and the formation of granulation tissue. Fibroblasts are also stimulated by growth factors produced by immune cells like macrophages and produce matrix metalloproteinases (MMPs) that break down the fibrin clot, facilitating cell migration^68^. Moreover, ECM molecules like fibronectin, hyaluronic acid, proteoglycans, and collagen (mostly type III) replace the fibrin clot with a new temporary matrix that facilitates keratinocyte migration, essential for re-epithelialization^69^. The production of collagen by fibroblasts during wound healing is crucial for maintaining structural integrity. Histologically, collagen synthesis can be assessed using Crossman’s trichrome staining, which differentiates collagen types by color: Type I collagen appears reddish orange, while Type III collagen is yellowish green. This staining technique helps visualize collagen fibers throughout the healing process^70^.

Immunohistochemistry findings further demonstrated that the lyophilized AM group exhibited increased levels of VEGF expression than the other groups. VEGF was expressed in the microvessels of the granulation tissue, as well as in activated fibroblasts and inflammatory cells such as mast cells, and macrophages, during the first week after wound induction^71–73^. As a crucial component in promoting capillary permeability and assisting in tissue regeneration, VEGF expression is also present in the tunica media during the healing response. Smooth muscle cells (SMCs) can be identified by staining blood vessel tunica media cells with VEGF. SMCs play a key role in wound healing^74,75^. Cells produce VEGF, a signaling protein that plays a crucial role in angiogenesis by stimulating the formation of blood vessels^76^. We demonstrated that VEGF was expressed in the blood vessels and in the basal stem cells of the epidermis. However, VEGF expression was sharply upregulated in response to cutaneous injury. The development of new blood vessels at the site of damage is associated with excessive VEGF transcription^77^. The levels of many proinflammatory cytokines, including IL1 and IL6, which are elevated in the early stages of wound healing, upregulate the expression of VEGF^78^. The influx of inflammatory cells into the site of damage, the synchronization of vascular permeability, and endothelial cell proliferation are all aspects of the role of VEGF in wound healing^79^.

However, despite the promising results, there are limitations to this study that should be acknowledged. The sample size was relatively small, so future studies with larger sample sizes are needed to confirm these results. Another limitation is the short duration of the study; long-term effects and the durability of the regenerated tissue were not assessed. Future research should also focus on long-term outcomes to ensure that the benefits of using amniotic membranes are sustained over time.

## Conclusion

Amniotic membrane offers numerous benefits, including availability, low cost, simple isolation, antibacterial properties, and low immunogenicity. These properties make it an ideal biomaterial for tissue engineering and regenerative medicine applications, particularly in engineered skin and soft tissue reconstruction. The biological properties and composition of amniotic membrane make it a promising candidate for wound treatment and tissue engineering. Further research into preservation methods and antibacterial effects could enhance its clinical utility in treating various medical conditions.

## Electronic supplementary material

Below is the link to the electronic supplementary material.

Supplementary Material 1

## Data Availability

The data that support the findings of this study are available on request from the corresponding author, upon reasonable request.
