# Supplementary material for: Efficacy of xenogeneic fresh and lyophilized amniotic membranes on the healing of experimentally induced full-thickness skin wounds in dogs
Source: Sci Rep. 2025 May 4;15:15605. doi: 10.1038/s41598-025-95023-9 (PMC12050321; doi:10.1038/s41598-025-95023-9)
Supplement: Supplementary file 1 — Supplementary Material 1 [file 41598_2025_95023_MOESM1_ESM.docx]

**List of abbreviations:**

AM: Amniotic membranes; bFGF: Basic fibroblast growth factor; DMEM: Dulbecco's modified Eagle Medium; EGF: Epidermal growth factor; ECM: Extracellular matrix; FBS: Fetal bovine serum; GAGs: Glycosaminoglycans, H&E: Hematoxylin and eosin; SEM: Scanning electron microscopy; TGF: Transforming growth factor; VEGF: Vascular endothelial growth factor.

**Supplementary Figures**


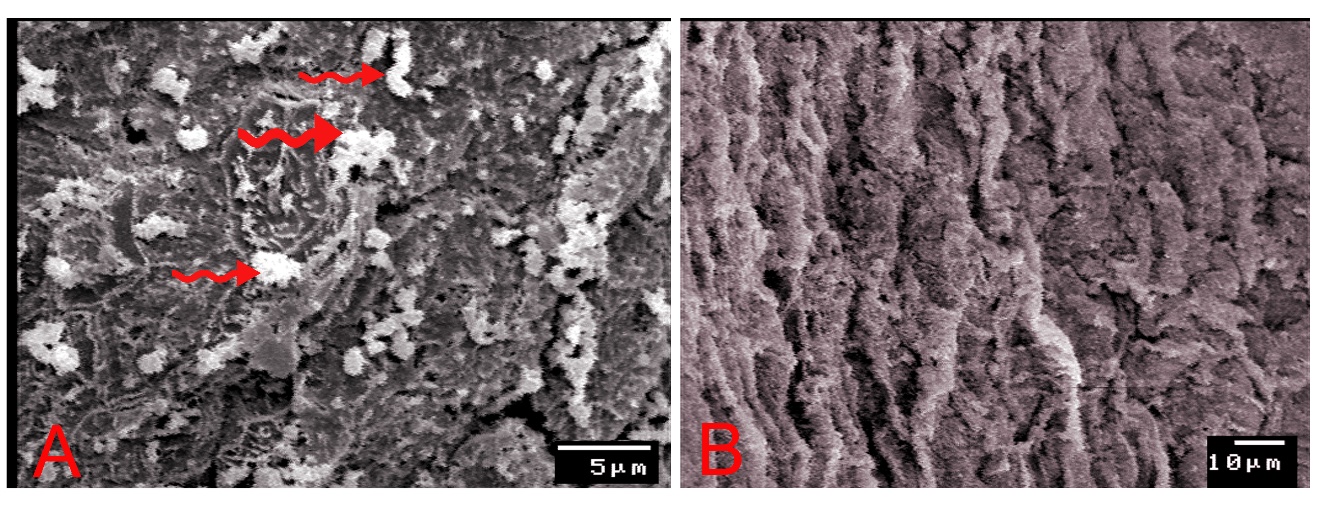


**Supplementary Fig 1. SEM images show fresh AM**. A: SEM revealed a monolayer with less distinct borders and a surface coated with microvilli and secretions on the apical side of the cells (wavy arrows). B: Collagenous fiber bundles were observed in the stroma of the fresh AM.


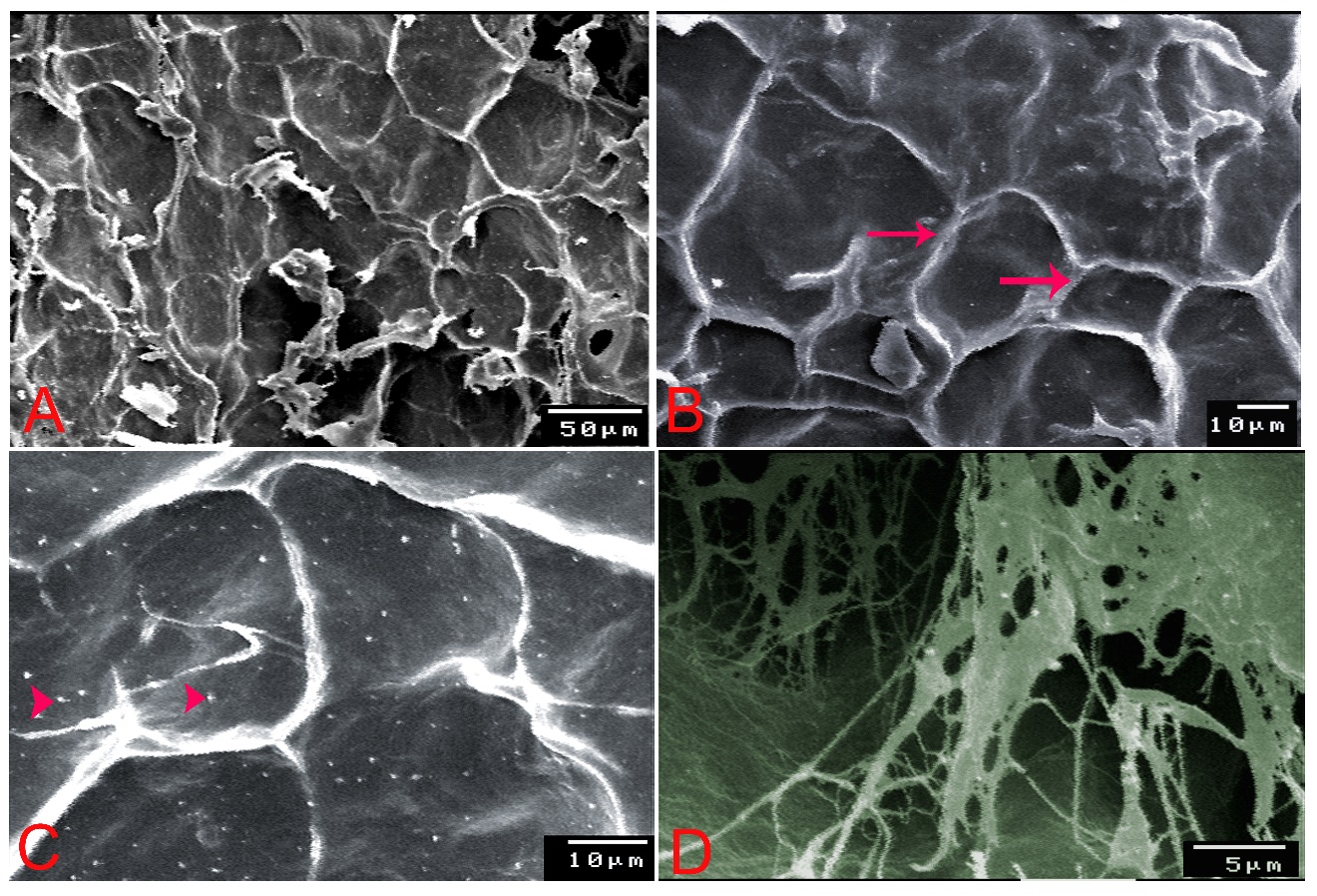


**Supplementary Fig 2. SEM images show lyophilized AM**. A-C: SEM revealed a well-defined apical border (arrows) with few microvilli (arrowheads) and no apical secretions. D: Network of multidirectional reticular fibers that made up the stroma.


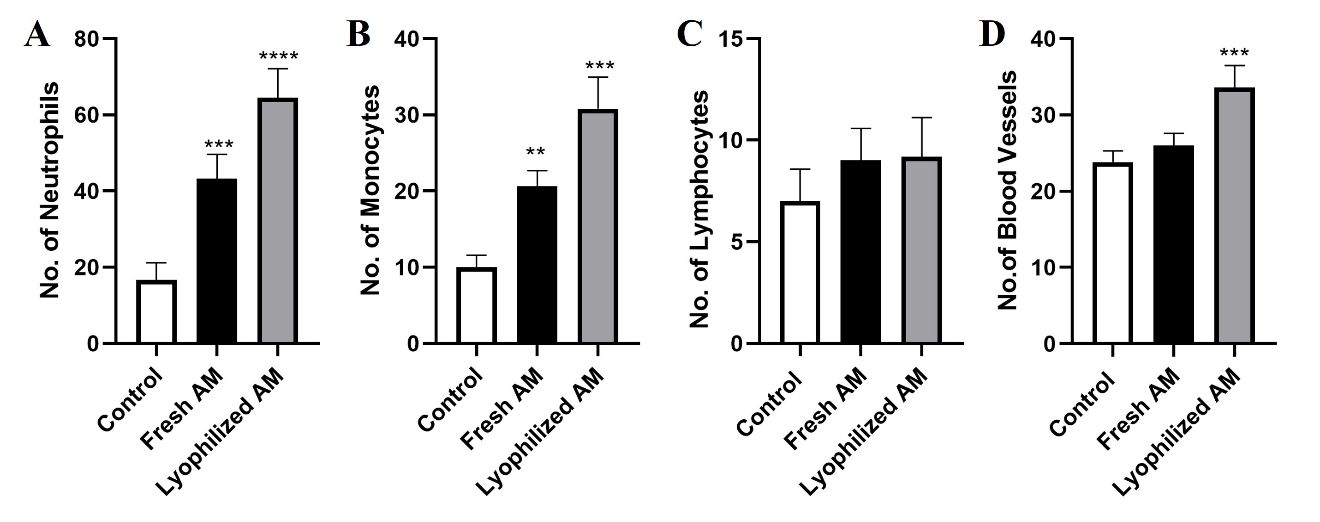


**Supplementary Fig 3.** Graphs show the number of neutrophils (A), monocytes (B), lymphocytes (C), and newly formed blood vessels (D) in the wound area of the different groups 1 week after wound induction. The results were compared to the control group, and differences were evaluated using t-test. **p* < 0.05; *** *p* < 0.001.


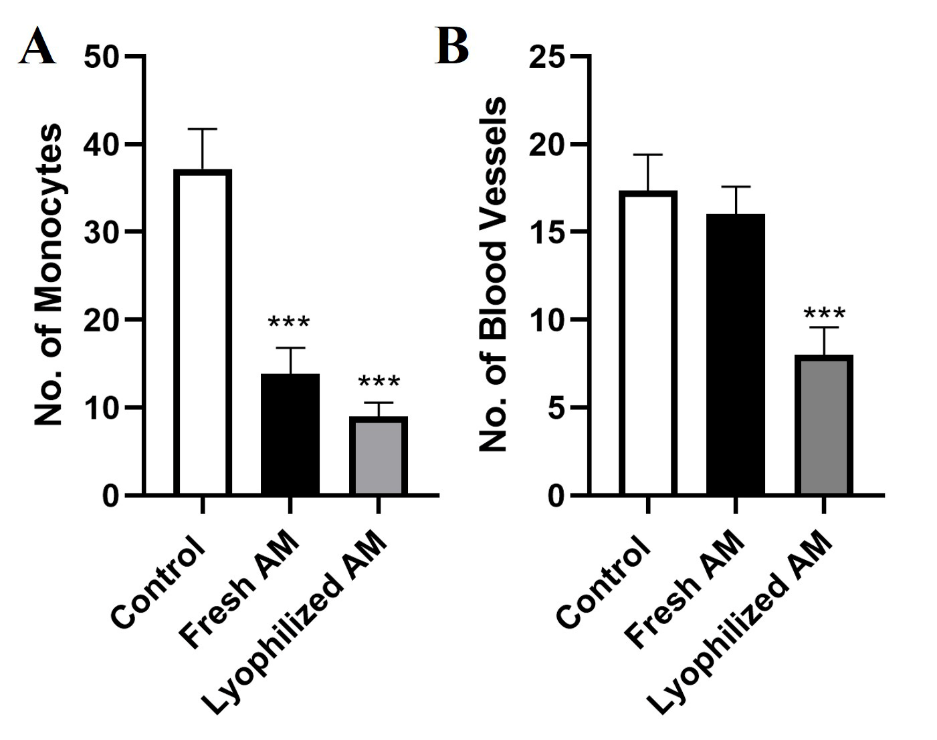


**Supplementary Fig 4.** Graphs show the number of monocytes (A) and newly formed blood vessels (B) in the wound area of the different groups 3 weeks after wound induction. The results were compared to the control group, and differences were evaluated using t-test. * *p* < 0.05; *** *p* < 0.001.
